# Metanephric stromal tumor in an adult with *PDGFRA* mutation: a case report

**DOI:** 10.1186/s13000-023-01372-2

**Published:** 2023-07-15

**Authors:** Sanjun Guo, Huan Qian, Hong Zhu, Yue Yang, Xudan Yang, Huajun Sun

**Affiliations:** 1 Department of Pathology, Sichuan Academy of Medical Sciences & Sichuan Provincial People’s Hospital, Chinese Academy of Sciences Sichuan Translational Medicine Research Hospital, Chengdu, 610072 China; 2 Department of Pathology, The First People’s Hospital of Guangyuan, Guangyuan, Sichuan 628017 China; 3 Department of Pathology, Sichuan Provincial People’s Hospital, University of Electronic Science and Technology of China, Chengdu, 611731 China

**Keywords:** Metanephric stromal tumor, Adult, *BRAF* V600E mutation, *PDGFRA* mutation, Small diameter tumor

## Abstract

**Background:**

Metanephric stromal tumors (MST) are rare benign renal tumors that mainly occur in infants and children. Approximately 72% of MST in children have the B-Raf proto-oncogene serine/threonine kinase *(BRAF)* V600E mutation. To date, only five cases of adult MSTs have been reported and no clear genetic alterations have been found.

**Case presentation:**

We report a case of MST in a 45-year-old woman who complained of left lower back pain for a week, accompanied by hypertension (150/79 mmHg). Magnetic resonance imaging (MRI) showed an abnormally enhanced nodule (1.1 cm in the middle of the left kidney), which was histopathologically consistent with an MST. The *BRAF* V600E mutation was not detected in tumor cells using PCR and next-generation sequencing (NGS). However, a platelet-derived growth factor receptor alpha (*PDGFRA*) mutation was detected in this case using NGS. The patient showed no recurrence or metastasis nine months after partial nephrectomy, and her blood pressure was consistently normal.

**Conclusion:**

This is the first report of alterations in *PDGFRA* in MSTs. This result advances our knowledge of genetic variations in adult MSTs, which may have different gene alterations from MSTs in children.

## Introduction

Metanephric stromal tumors (MST) are rare benign renal tumors that have been identified in the last 20 years. It is prevalent in infants and children and is typically large [1]. The most common gene alteration in the MSTs of children (72%) is the B-Raf proto-oncogene serine/threonine kinase (*BRAF)* V600E mutation [1]. Although rare, adult MSTs have been reported. To the best of our knowledge, five cases of adult MSTs have been described over the world [2]. Among them, only one patient underwent genetic testing and showed no *BRAF* V600E mutation in the tumor [3]. Herein, we report a case of a small MST in a 45-year-old woman and explore its genetic changes using PCR and next-generation sequencing (NGS).

## Case presentation

**Fig. 1:**
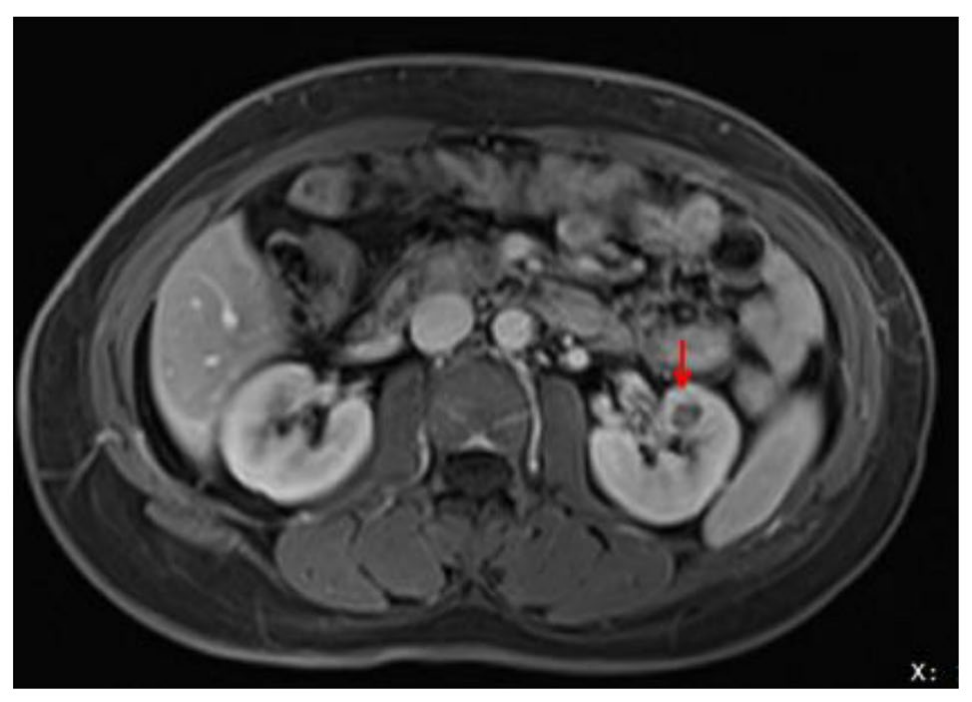
Magnetic resonance imaging (MRI) shows a mass in the left kidney (red arrow)

**Fig. 2:**
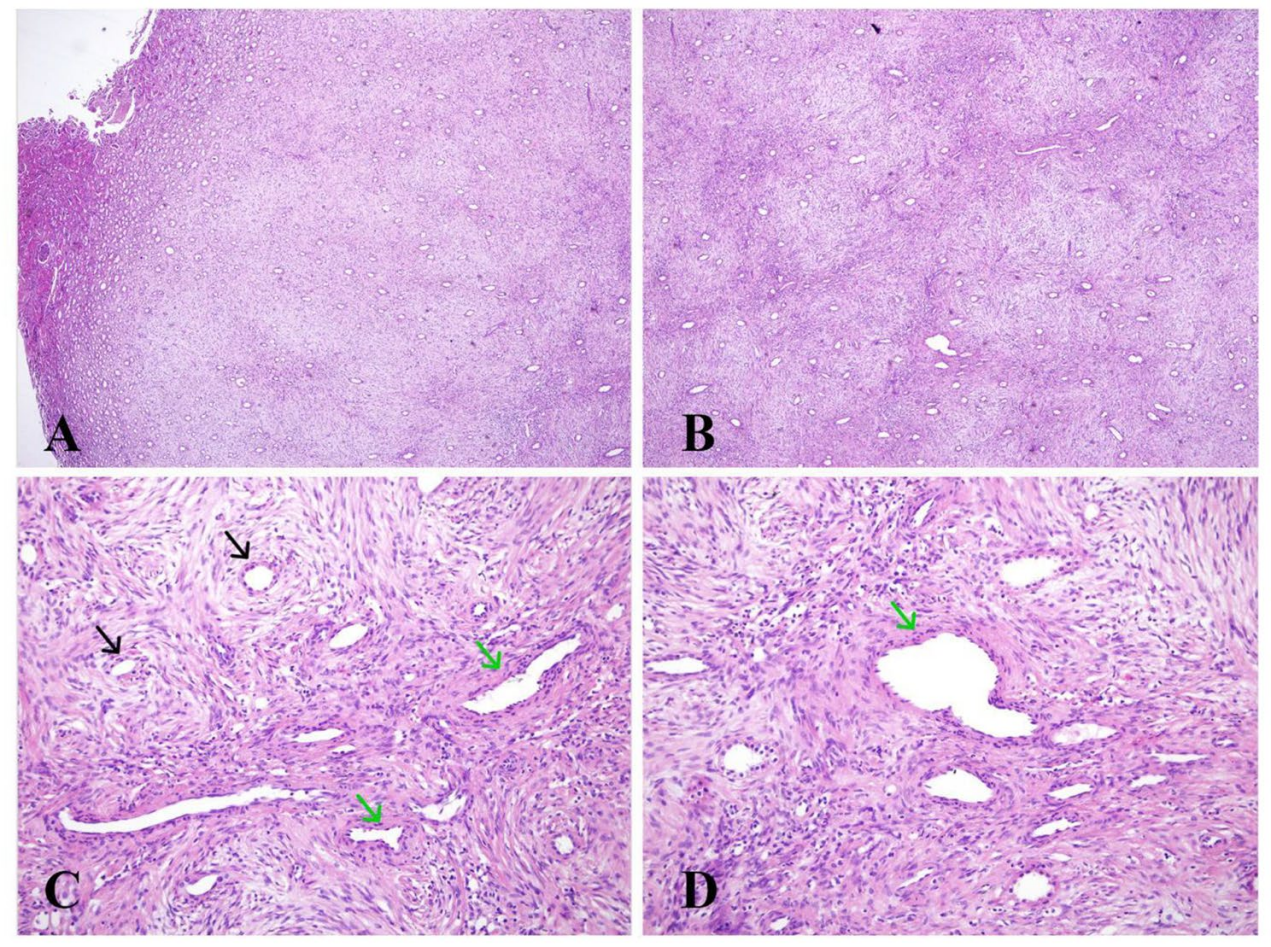
Pathological features of metanephric stromal tumor (MST). **A**: The MST was located in the medulla, with subtle infiltration into the adjacent renal parenchyma (Hematoxylin and eosin stain (HE), 4×). **B**: The MST shows a nodular appearance and interstitial myxoid degeneration (HE, 10×). **C**: The tumor cells form a slight “onion-skin” appearance surrounding entrapped renal tubules (black arrow) (HE, 20×). **C**–**D**: Dysplastic small vessels were observed (green arrow) (HE, 20×)

A 45-year-old woman was admitted to the Sichuan Provincial People’s Hospital complaining of left lower back pain for one week, accompanied by hypertension (150/79 mmHg). The patient had no previous history of malignancy or family history of renal tumors. Magnetic resonance imaging (MRI) revealed an abnormally enhanced nodule (1.1 cm) in the middle of the left kidney (Fig. 1). The patient underwent partial nephrectomy with robot-assisted surgery for tumor removal. Her blood pressure returned to normal (95/63 mmHg) 15 d postoperatively. Follow-up information was obtained through telephone consultations. The patient showed no recurrence or metastasis nine months after surgery, and the blood pressure was normal. Histological examination: Gross examination revealed a 1.2 cm mass, and the cut surface of the tumor was gray and solid, with no areas of hemorrhage, necrosis, or cystic change. Under low power field of the microscope, the tumor was unencapsulated, involved the renal medulla, and had subtly infiltrated the adjacent renal parenchyma (Fig. 2A). The tumor exhibited a slightly nodular appearance and the stroma showed myxoid degeneration (Fig. 2B). Under high power, the tumor cells were spindle- or star-shaped and exhibited thin, tapered, hyperchromatic nuclei, indistinct cytoplasmic extensions, and low mitotic activity (< 1/high power field (HPF)). The tumor cells showed slight concentric circular changes surrounding the entrapped renal tubules or vessels (Fig. 2C). Additionally, small dysplastic vessels were observed (Fig. 2C–D).

**Fig. 3:**
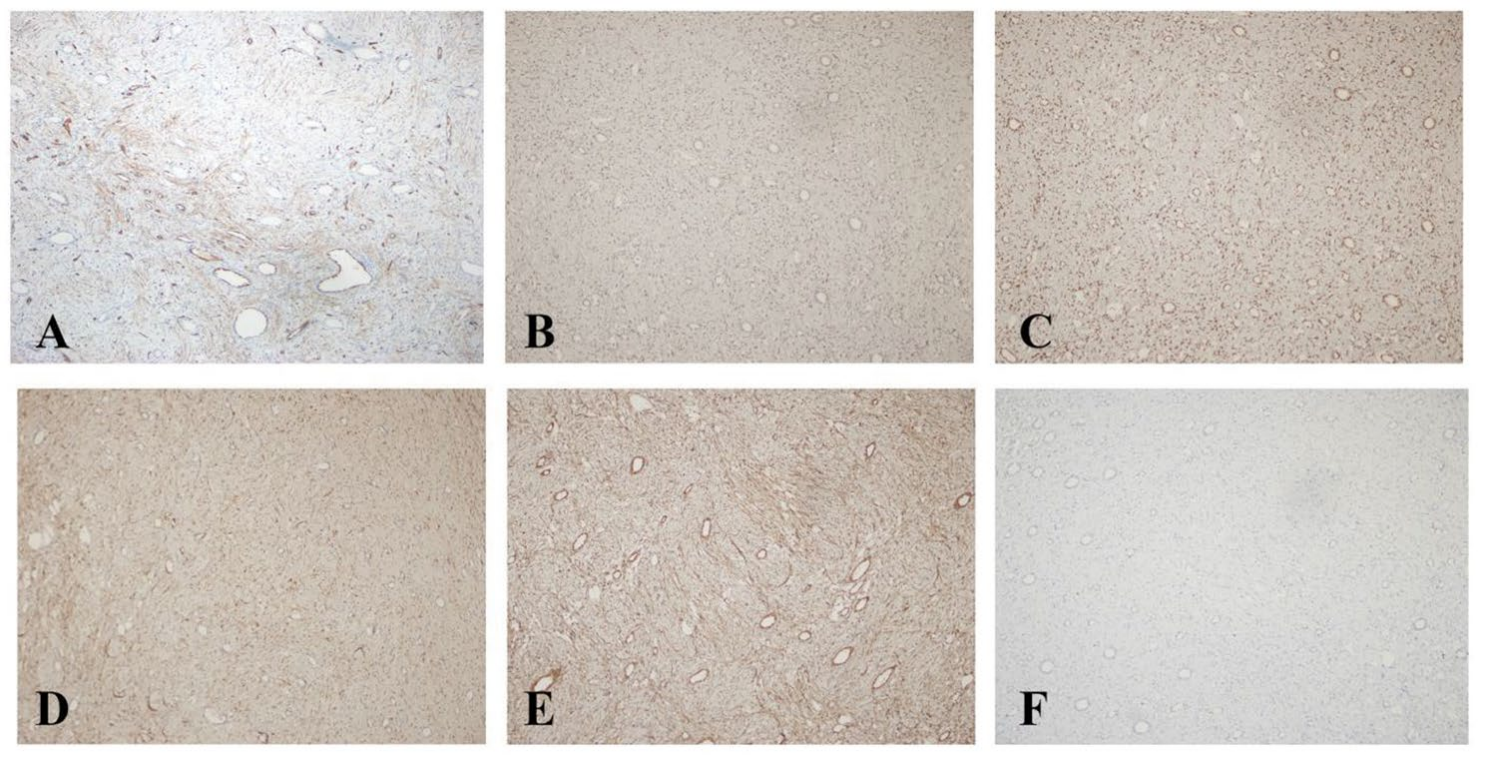
Immunohistochemical manifestations of MST. **A**: Immunohistochemistry (IHC) shows mottled positive expression for CD34 (20×). Positive expression of ER, PR, CD99, and Bcl-2 is shown in the order **B**–**E**(20×). **F**: WT-1 was negative in both tumors cells and the entrapped native renal tubules (20×)

Immunohistochemical manifestations: The tumor cells displayed a mottled immune response to CD34 (Fig. 3A) and diffused Vimentin expression. Additionally, the cells were positive for ER, PR, CD99, and Bcl-2 (Fig. 3B–E). Moreover, the tumor cells were negative for CD117, Dog-1, ALK, P63, Desmin, Calponin, SMA, GFAP, EMA, HMB45, MelanA, S-100, SOX10, CAIX, TSH, PRL, ACTH, GH, CD56, CgA, SSTR-2, Syn, and WT-1 (Fig. 3F) expression. In addition, the tumor cells had a low Ki-67 index and no INI-1 deletion. Entrapped native renal tubules were highlighted using PAX-8, CAM5.2, and PCK; however, they were negative for WT-1.

**Fig. 4:**
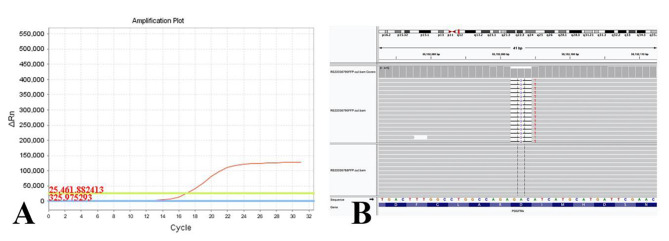
Molecular alterations of MST. **A**: B-Raf proto-oncogene serine/threonine kinase *(BRAF)* V600E mutation was not detected using PCR. **B**: Amino acids 842–843 of the platelet-derived growth factor receptor alpha (*PDGFRA*) gene were deleted, and base A was mutated to base T

Genetic analysis: Detection of the *BRAF* V600E gene was performed using PCR in paraffin sections of the tumor using the Human *BRAF* Gene V600E Mutation Detection Kit (Fluorescent PCR Method, AmoyDx, Xiamen, China). The results demonstrated that *BRAF* V600E was wild-type in the adult MST (Fig. 4A). Subsequently, NGS was performed on the tumor tissue, and normal renal tissue 2 cm from the tumor was used as a control for germline genetic detection. The results of NGS showed that the *BRAF* V600E mutation was not detected in either MST cells or normal renal tissues, which was consistent with the PCR results. However, we discovered a novel genetic variation in this adult MST. The NGS results of the tumor cells showed that amino acids 842–843 of the platelet-derived growth factor receptor alpha (*PDGFRA*) were deleted, and base A was mutated to base T, inducing the mutation of isoleucine to phenylalanine (Fig. 4B). Therefore, the tumor cells showed a systemic *PDGFRA* mutation; however, the corresponding germline mutation was not detected.

## Discussion

MST is a rare renal tumor that has been identified in the last 20 years. MSTs mainly occur during infancy and early childhood, and the youngest recorded patient was only 2 d old [4]. Owing to the rarity of MSTs, these tumors have mainly been discussed in case reports. To the best of our knowledge, only five of the 50 MSTs reported to date have been reported in adults [2]. The first adult MST was reported in 2002 by Bluebond in a 53-year-old woman [5]. The age at diagnosis of the five adult patients with MST was between 53 and 77 years, and the male-to-female ratio was 1:1.5. The most common genetic change in children with MST is the *BRAF* V600E mutation [1]. In five cases of adult MST, genetic testing was performed in only one case, and no *BRAF* V600E mutation was detected in the tumor [3]. Here, we have presented a case of MST in a 45-year-old woman who underwent genetic testing for the tumor.

Zhang et al. reviewed 47 cases of MST and found that 97.8% (46/47) of MSTs were located in the medulla, except for one in the renal cortex, with an average diameter of 5.7 cm and the smallest diameter of 2.5 cm [1, 6]. The tumor sizes in five cases of adult MSTs were approximately 2.5–21 cm [2, 5, 7, 8]. The tumor that we have reported was located in the renal medulla, with a diameter of 1.2 cm, which is the smallest reported diameter of an MST. Typical histological features of MSTs include spindled or stellate tumor cells and a nodular appearance with concentric or “onion-skin” cuffing surrounding the entrapped renal tubules or vessels. Heterologous differentiation of glial tissue, adipose tissue, cartilage, and squamous epithelia has also been reported in several cases. The diagnostic significance of immunohistochemistry (IHC) is the patchy positivity for CD34 [1, 2, 6, 9–12]. Our tumor exhibited a slight nodular appearance, with “onion-skin” appearance surrounding wrapped tubules and vessels, and dysplastic vessels. Heterologous differentiation was not observed in the adult MST. The IHC results showed that the tumor cells were mottled and positive for CD34. Immunoreactivities for ER and PR were also detected, and this phenomenon was consistent with that reported by Chaudhri [2].

According to the World Health Organization (WHO) Classification of Tumors of the Urinary System and Male Genital Organs (5th edition), MST, metanephric adenoma, and metanephric adenofibroma are classified as metanephrogenic tumors because of their similar morphologies and tumor properties [13]. In the pathological diagnosis, MST must be distinguished from metanephric adenoma and metanephric adenofibroma. Metanephric adenomas are tumors of epithelial origin, and the tumor cells are positive for WT-1. Metanephric adenofibroma are tumors of mixed epithelial and mesenchymal origin and the epithelial component expresses WT-1, whereas the invaginated tubules in MST are negative for WT-1. In conclusion, this adult MST could be identified from mimic tumors using histology and IHC.

Most MSTs are asymptomatic (53.2%), whereas some patients experience abdominal discomfort or pain (21.3%), urinary tract infections (6.4%), hypertension (10.6%), and hematuria (17.0%) [1]. Histologically, nearly 27.7% of the MSTs showed juxtaglomerular cell hyperplasia [1]. Some researchers have hypothesized that the cortical glomeruli entrapped by MST tumor cells induce hyperplasia of juxtaglomerular cells, subsequent secretion of renin, and cause hypertension [5]. Although the clinical progression of our patient demonstrated that her hypertensive symptoms were closely related to MST, juxtaglomerular cell hyperplasia was not observed in the tumor.

The most common molecular alteration in pediatric MSTs is the *BRAF* V600E mutation, with an occurrence rate of 72% [1]. Marsden et al. collected 17 pediatric MSTs, 22 congenital mesoblastic nephromas (CMNs), and six ossifying renal tumors of infancy for testing *BRAF* exon 15 using PCR amplification and Sanger dideoxy sequencing methods. Their results showed that the *BRAF* V600E mutation was found in 11/17 (65%) cases of pediatric MSTs, and all other renal stromal tumors tested were negative for the *BRAF* exon 15 mutation [14]. In addition, a few case reports have described *BRAF* V600E mutations in pediatric MSTs [3, 15]. Furthermore, Toutain et al. found that the terminal long arm of chromosome 17 was rearranged in the MST of a three-year-old boy [16]. However, no chromosomal abnormalities were reported in the subsequent cases. Because of the extremely raritye cases, there have been no clear reports of gene alterations in adult MSTs. Among the five reported cases of adult MSTs, only one patient underwent genetic testing. A study by Pedram included six children and one adult with MSTs. They demonstrated that the *BRAF* V600E mutation was detected in MST tissues from all six children, but was not detected in the MST tissue from the adult [3]. Additionally, we did not find the *BRAF* V600E mutation in our adult MST using PCR and NGS, which was consistent with the results of the study by Pedram. However, the tumor cells exhibited systemic *PDGFRA* mutations in our study. This is the first report of an MST with a *PDGFRA* mutation. The results of the study by Pedram et al. and those of our case revealed that MSTs in adults may have genetic variations different from those in children. Due to the scarcity of genetic studies on adult MSTs, this conclusion requires further verification by genetic sequencing of more adult MSTs.

*PDGFRA* encodes a membrane surface tyrosine kinase receptor that binds to platelet-derived growth factors (PDGFs) and forms a homodimer or heterodimer, thereby mediating many biological processes, including organ development, wound healing, cell proliferation, angiogenesis, and differentiation [17, 18]. Therefore, PDGFRA plays an important role in tumor progression. *PDGFRA* mutations are associated with gastrointestinal stromal tumors (GIST) [19, 20]. The Cancer Genome Atlas(TCGA) data show that *PDGFRA* mutations and amplifications are also common in malignant tumors, such as glioblastoma [21], melanoma [22, 23], non-small cell lung cancer [24], and hematologic malignancies [25].

*BRAF* is a serine/threonine protein kinase of the *R*af family that belongs to the Ras-Raf-MAPK signaling pathway, which is triggered by several receptor tyrosine kinases (TKs) such as *KIT* and *PDGFRA* [26]. The Ras-Raf-MAPK signaling pathway modulates cell proliferation, survival, differentiation, metabolism, and migration. Approximately 8% of the gastrointestinal stromal tumors devoid of *KIT*/ *PDGFRA* mutations bear the *BRAF* mutation [27]. Therefore, *BRAF* V600E mutation may play a compensatory role in GIST pathogenesis [28]. Recent studies have revealed that *BRAF* mutations can occur concomitantly with *KIT*/*PDGFRA* mutations [29]. Previous studies have found that the most common gene mutation in MSTs was the *BRAF* V600E mutation; whereas, in our study, a *PDGFRA* gene mutation was found for the first time. Because *BRAF* and *PDGFRA* are located differently in the RAS-RAF-MAPK signaling pathway, we hypothesized that these two mutations may be mutually alternative or concurrent during MST tumorigenesis.

However, to our knowledge, this is the first report of a *PDGFRA* mutation in an MST. Pathologically, the studied tumor had a typical MST morphology and negative expression of CD117 and DOG-1, as observed using IHC. Clinically, the patient showed symptoms of hypertension that were highly associated with the tumor; furthermore, she had no history of GIST. Therefore, we excluded the possibility of metastatic GIST in the kidney and confirmed the diagnosis of adult MST.

Most patients with MST show no recurrence or metastasis at the end of follow-up after total or partial nephrectomy and nephroureterectomy [1]. The conventional treatment for MSTs is laparotomy, whereas our patient underwent robot-assisted partial nephrectomy. Recent studies have shown that the specific advantages of robot-assisted surgery include minimal invasiveness, alleviation of postoperative pain, reduced bleeding, shortened hospital stay, and improved postoperative recovery [30–33]. In adult urological oncology, partial nephrectomy is an option for the treatment of small renal masses, and robotics has become commonplace. Robot-assisted partial nephrectomy was the preferred choice because of the small diameter of the MST. To the best of our knowledge, this is the first patient with MST who was treated with robot-assisted partial nephrectomy and showed a good prognosis, similar to that of laparotomy. Future applications of robot-assisted surgery in larger or pediatric MSTs are worth exploring.

## Conclusion

In conclusion, we have presented a rare case of MST with the smallest diameter reported to date in an adult patient. A new genetic alteration, the *PDGFRA* mutation, was found in the adult MST, which has not been reported previously. This case broadens the clinicopathological knowledge and spectrum of genetic variations in MSTs in adults. MSTs in adults may have genetic alterations that are different from those in children.

## Data Availability

The data that support the findings of this study are available from the Sichuan Provincial People’s Hospital, but restrictions apply to the availability of these data, which were used under the license for the current study and so are not publicly available. However, data are available from the authors upon reasonable request and with permission from the Sichuan Provincial People’s Hospital.
